# Estimating winning percentage of the fourth quarter in close NBA games using Bayesian logistic modeling

**DOI:** 10.3389/fpsyg.2024.1383084

**Published:** 2024-05-03

**Authors:** Feng Wang, Guohua Zheng, Hua Li

**Affiliations:** ^1^School of Economics and Management, Shanghai University of Sport, Shanghai, China; ^2^School of Physical Education, Xiangnan University, Chenzhou, China; ^3^Department of Physical Education, Hohai University, Nanjing, China

**Keywords:** game performance, pace of basketball games, Bayesian logistic regression, basketball games, statistical methods

## Abstract

This study examined the fourth quarters in the close games in the regular NBA games in the last decade, ranging from the 2013–14 season to the 2022–2023 season. A close game is categorically defined by a scenario where the point differential is confined within a 10-point margin at the onset of the fourth quarter and narrows further to a 5-point disparity by the end of the game. In total, 2,295 close games were identified in this study. Advanced game statistics, including offensive rate, defensive rate, assistance ratio, pace of game, and true shooting percentage, etc., are obtained from the NBA box scores using a python script. Understanding key factors that determine the outcome of the basketball games is critical, as such can be used to develop predictive models for coaches to design game strategies. This study developed a Bayesian Logistic Modeling approach to estimate the winning probability of a basketball team in the fourth quarter, using the pace of the last quarter and a team’s shooting percentage. The accuracy of the model is used to evaluate if the model can correctly classify game outcome based on the identified game statistics in the fourth quarter of a game. The binary outcome of the close game is modeled as a Bernoulli distribution. Results reveal that the True Positive Rate and False Positive Rate is 0.93 and 0.07, respectively. Insights from this study can be used to help design coaching strategies in basketball games, illuminating potential tactical pivots that could tilt the game in their favor.

## 1 Introduction

In professional basketball, the importance of the fourth quarter is paramount, often determining the outcome of closely contested games (Gomez et al., 2016). It’s a period characterized by heightened intensity, where the amalgamation of skill, strategy, and mental fortitude is put to the ultimate test (Taylor and Taylor, 1997). Key performance indicators (KPI) found important in other studies in the literature that affect match outcomes would be also affect the fourth quarter of a basketball game.

Factors that determine game outcomes can be categories as two: internal variables and external variables in Huyghe et al. (2022). Similarly, Player Gomez et al. (2016) identify those as situational variables (starting quarter score, game location and quality of opposition) and technical-tactical variables (game situation, defense type, shot type). The internal variables are related to basketball players’ performance, including field goal percentage, offensive and defensive rebound, average fouls and average steals, assistance, and pace of the game (Bar-Eli and Tractinsky, 2000; Melnick, 2001; Teramoto and Cross, 2010; Arkes and Martinez, 2011; Mikolajec et al., 2013). The external factors include change of team management or coaches, game location (home or away), and traveling and back-to-back games (Price et al., 2010; Martínez and Caudill, 2013; Nutting and Price, 2015; Esteves et al., 2021).

There are unique factors that could be influential for the fourth quarter of a basketball game, given every pass, shot, and defensive play is amplified, echoing the profound impact of individual and collective actions during these decisive minutes. First, Strategic adaptability emerges as a cornerstone in the fourth quarter. Coaches are compelled to make real-time adjustments, drawing from their observation and analysis of the game’s unfolding dynamics (Franks and Miller, 1991). Players, in turn, are tasked with the immediate and adept application of these adjustments, a scenario that underscores the importance of experience and team synergy. Basketball players’ ability to focus and make decisive plays underscores the intricate blend of psychological and physical prowess that’s activated in the fourth quarter (O'Donoghue, 2010). Second, fatigue plays an important role. The fatigue effect, which refers to the fact that with the progression of the game, intense offense and defense burn an increased amount of energy for basketball players, often results in reduced activity and performance (Kordyaka et al., 2022). A study by McInnes et al. (1995) investigated the role of fatigue, revealing that players exhibit a marked decrease in both shooting accuracy and overall effectiveness due to physical exhaustion incurred throughout the game. García et al. (2020) examined physical demands, including basketball players’ peak velocity, total distance covered, high-speed running and compared those variables between game quarters. The authors found that there was an overall decrease in all variables between the first and fourth quarter during competition. Similarly, Wang and Zheng (2022a,b) examined NBA games in the last decade and found that within a quarter of the game, the field goal accuracy reduces as the games progress to the last 3-min segment within a quarter.

In recent years, the sports analytics research community has increasingly embraced Bayesian methods. Reich et al. (2006) innovated a spatial hierarchical model, offering nuanced evaluations of a basketball player’s shooting accuracy across various zones of the court. Hu et al. (2021) pioneered a Bayesian method crafted to scrutinize the heterogenous structure of shot selection among basketball players, enhancing the depth of understanding in players’ shooting decisions. Wang and Zheng (2022a,b) deployed a hierarchical model to scrutinize the correlation between positional differences and field accuracy. Their study delineated the nuanced variations in accuracy among players occupying different positions, bridging a critical knowledge gap. Nevertheless, the application of the Bayesian logistic model specifically to the analysis of performances in the fourth quarter of basketball games remains uncharted territory. The unexplored application of Bayesian models in this specific context promises a frontier for novel insights and enhanced predictive analytics in the realm of professional basketball.

This study aims to examine close basketball games in the NBA in the past decade and identify team statistics that play an important role in determining the outcome of the game. In addition, a robust Bayesian logistic modeling method is proposed to estimate a team’s winning probability, based on the pace of the game and true shooting percentage of the team. The robust Bayesian logistic model, rather than the standard Bayesian logistic model, is chosen given its benefit of minimizing the effect of extreme values on regression results (Kruschke, 2015). The proposed model is tested for a professional basketball team. This study introduces a nuanced perspective that synergizes quantitative analysis with practical, actionable insights, enhancing the strategic depth in understanding and interpreting the complex, multifaceted nature of close basketball contests in the NBA.

## 2 Materials and methods

### 2.1 Data

Relevant game statistics from close NBA games in the past decade, ranging from 2013–14 season to 2022–23 season, were obtained for this analysis.^1^ A closely contested game is characterized by a margin of fewer than 10 points as the fourth quarter begins, and the ultimate victor emerges with a lead of less than 5 points by the end of the fourth quarter. To facilitate data retrieving, the NBA application programming interface (API)^2^ was used to download data from the NBA’s website. The NBA API for data retrieval is a set of online protocols and tools, namely endpoints, for obtaining various types of data related to the National Basketball Association. It allows developers, data analysts, and basketball enthusiasts to fetch real-time or historical data about players, teams, games, statistics, and other NBA-related information. In total, three original datasets were collected from three endpoints, including the LeagueGameFinder, PlayByPlayV2, and BoxScoreAdvancedV2. The endpoint LeagueGameFinder provides game relevant statistics and win/lose team for each game. The endpoint PlayByPlayV2 provides play-by-play of all the games and lists the point difference at the beginning and end of each quarter. The BoxScoreAdvancedV2 endpoint summarize team statistics for the fourth quarter, including offensive and defensive rating, pace of the game, assistance percentage, and true shooting percentage, etc.

In acknowledgment of the distinct variances in the dynamics between regular-season games and playoff contests, this study specifically curates a dataset encompassing only regular-season encounters wherein the winning team is decisively ascertained at the conclusion of the fourth quarter. The methodological approach to data filtration involves a meticulous examination of the point differential between the competing teams both at the initiation and termination of the fourth quarter. For the purposes of this analysis, a ‘close game’ is categorically defined by a scenario where the point differential is confined within a 10-point margin at the onset of the fourth quarter and narrows further to a 5-point disparity by the close of the game. This criterion ensures a focus on contests characterized by competitive equilibrium, facilitating a nuanced exploration of strategies and performance under pressurized, closely contested game conditions.

**Figure 1:**
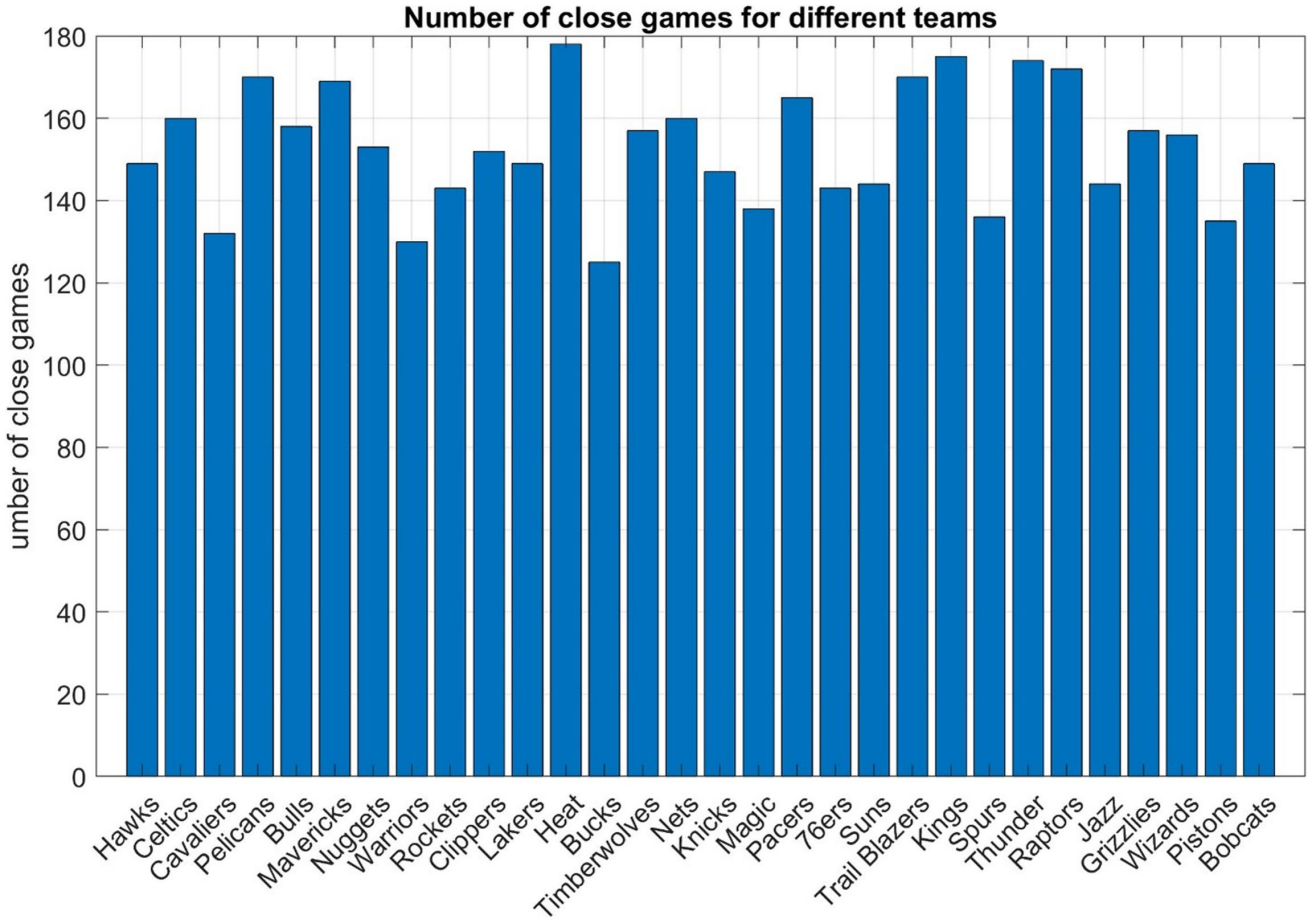
Close matchups in the regular seasons from the 2013–14 to the 2022–23 season. In total, 2,295 games (nearly 20%) met the criteria such that a margin of fewer than 10 points as the fourth quarter begins, and the ultimate victor emerges with a lead of less than 5 points by the end of the fourth quarter.

In total, 2,295 games (nearly 19%) met the criteria and Figure 1 shows such games for each NBA team. For each team, the close games are divided into winning and losing ones for further analysis described in sections below.

### 2.2 Robust Bayesian logistic modeling

**Figure 2:**
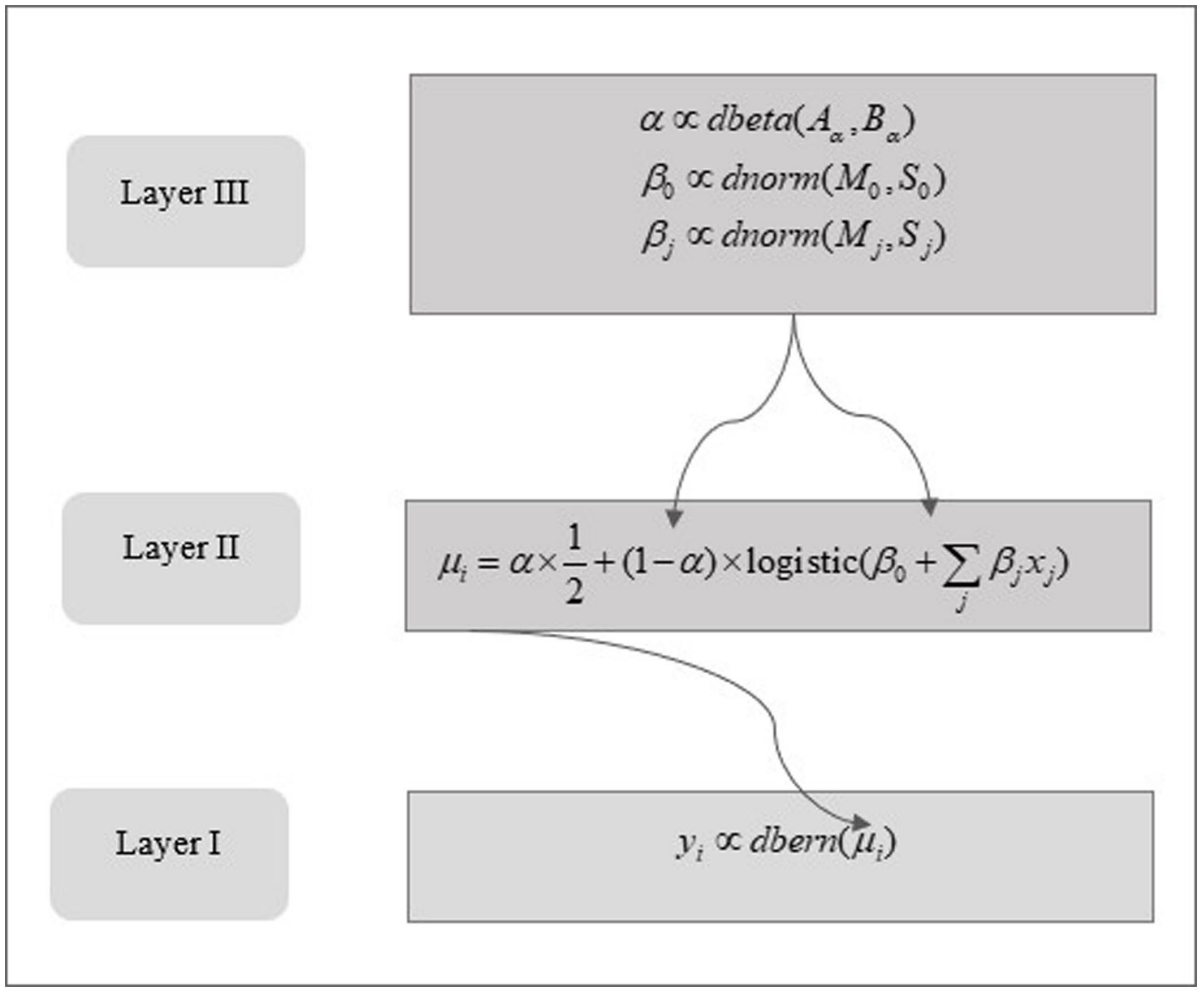
The structure of a Bayesian logistic model to predict probabilistic game outcome.

Figure 2 below shows the structural framework of the robust Bayesian Logistic Regression model, highlighting the hierarchical arrangement intrinsic to the proposed approach.

The notations and expressions illustrated in Figure 2 depict the model parameters and their associated probability distribution functions. In this context, *y*_i_ denotes a binary outcome. In the preprocessing stage for the Bayesian model *y*_i_ values are binarized to 0 or 1. For example, a value of 1 is assigned to *y*_i_ in instances where a team secures victory in a close game; otherwise, a value of 0 is allocated. The random variable *y*_i_ is postulated to adhere to the Bernoulli distribution, characterized by a parameter *μ*_i_ as defined in Equation (1) and illustrated in the initial layer of the model structure in Figure 2. The parameter *μ*_i_ is formulated in Equation (2). The first term is a “guessing” parameter, symbolizing the likelihood that the binary outcome, specifically 0 or 1, is an outcome of a Bernoulli random process with a parameter value of 0.5. The latter term encompasses a linear combination of predictors, including the pace of the fourth quarter and true shooting percentage, integrated with intercept parameters β_0_ and slope parameters β_j_, where *j* indexes the array of predictors. This expression in Equation (2) facilitates the incorporation of outliers, addressing anomalies inadequately represented by the established relationship between predictors and predictand.

Practically, Equation (2) can be interpreted as a weighted combination of a predetermined random process — exemplified by a Bernoulli process with a parameter value of 0.5 — and a logistic function. The transition to a conventional logistic function is realized when the “guessing” parameter is nullified, while the maximization of this parameter transforms Equation (2) into a descriptor for a random Bernoulli process.


(1) $$y_{i}\sim Bernoulli\left(\mu_{i}\right)$$

(2) $$\mu_{i}=\alpha \times \frac{1}{2}+\left(1-\alpha \right)\times \mathrm{logistic}\left(\beta_{0}+\underset{j}{\sum }\beta_{j}x_{j,i}\right)$$

(3) $$\beta_{0}\sim N\left(M_{0},S_{0}\right)$$

(4) $$\beta_{j}\sim N\left(M_{j},S_{j}\right)$$

(5) α~dbeta(1,100)

In total, there are four parameters in the hierarchical Bayesian model, including α, $\beta_{0}$,$\beta_{1}$, and$\beta_{2}$, when pace of the game and true shooting percentage are used in the logistic function. Prior distributions of the model parameters are shown in Equations (3)–(5). Specifically, the guessing parameter α is assumed to follow a Beta distribution characterized by a small mean value. This choice of prior distribution is motivated by the desire to impart a relatively larger weight to the logistic function, The intercept parameter $\beta_{0}$ and slope parameters $\beta_{1}$ and $\beta_{2}$ are assumed to follow the normal distribution.

The posterior distribution of model parameters was derived by employing the Markov Chain Monte Carlo (MCMC) technique, utilizing the Gibbs sampling algorithm. The implementation of this approach was facilitated through the utilization of the “Just Another Gibbs Sampler” (JAGS) package, employing a total of four distinct chains. Each individual chain was comprised of 7,500 iterations and the initial 1,000 iterations were designated as the burn-in period, serving to stabilize the chains and ensure convergence. To enhance the efficacy of the MCMC algorithm, a preprocessing step was undertaken, involving the standardization of predictors. Both the pace of the game and the true shooting percentage were standardized first before its use in the modeling process (Kruschke, 2015).

### 2.3 Evaluation metrics

Several evaluation metrics were employed in this study to assess the quality of the probabilistic categorical forecasts. The first metric employed is the Brier Score (BS), as shown in Equation (6).


(6) $$BS=\frac{1}{J}\overset{J}{\underset{j=1}{\sum }}{\left(p_{j}-o_{j}\right)}^{2}$$

Where $p_{j}$ is the predictive probability of the occurrence and $o_{j}$refers to the observed outcome. In this study, $o_{j}$ is 1 if the team wins the close game and is zero otherwise. The predictive probability of a team winning a game is calculated based on the model described in the last section. Notation *J* represents the total number of games used for model validation.

The second metric employed in our evaluation is the Brier Skill Score (BSS). This allows a comparative assessment of the Brier score obtained from our proposed method with other forecasting approaches, such as the winning probability derived from team records. The winning probability derived from team records serves as the reference model in this context. The mathematical formulation for computing the BSS is shown in Equation (7). The Brier Skill Score ranges from −∞ to 1, with a perfect score of 1 indicating that the predictive model has perfect skill, meaning it is completely accurate and outperforms the reference forecast. A score of 0 suggests that the forecast model performs no better than the reference prediction, and negative values indicate that the model performs worse than the reference prediction.


(7) $$BSS=1-\frac{BS}{BS_{0}}$$

Two additional metrics considered in this study are the True Positive Rate and the False Positive Rate, as shown in Equations (8) and (9), respectively.


(8) $$TP=\frac{n}{m}$$

(9) $$FP=\frac{v}{w}$$

In Eqn. (8), the variable ‘*m*’ signifies the total count of instances in which the modeling team emerges victorious in close games. Within this set of ‘*m*’ close games, ‘*n*’ represents the subset of cases where the predictive model correctly forecasts a victory for the team. Similarly, in Eqn. (9), the variable ‘*w*’ denotes the total number of occurrences in which the modeling team incurs losses in close games. Within this set of ‘*w*’ close games, ‘*v*’ designates the subset of instances where the predictive model erroneously predicts a victory for the team.

## 3 Results

### 3.1 Descriptive statistics

**Figure 3:**
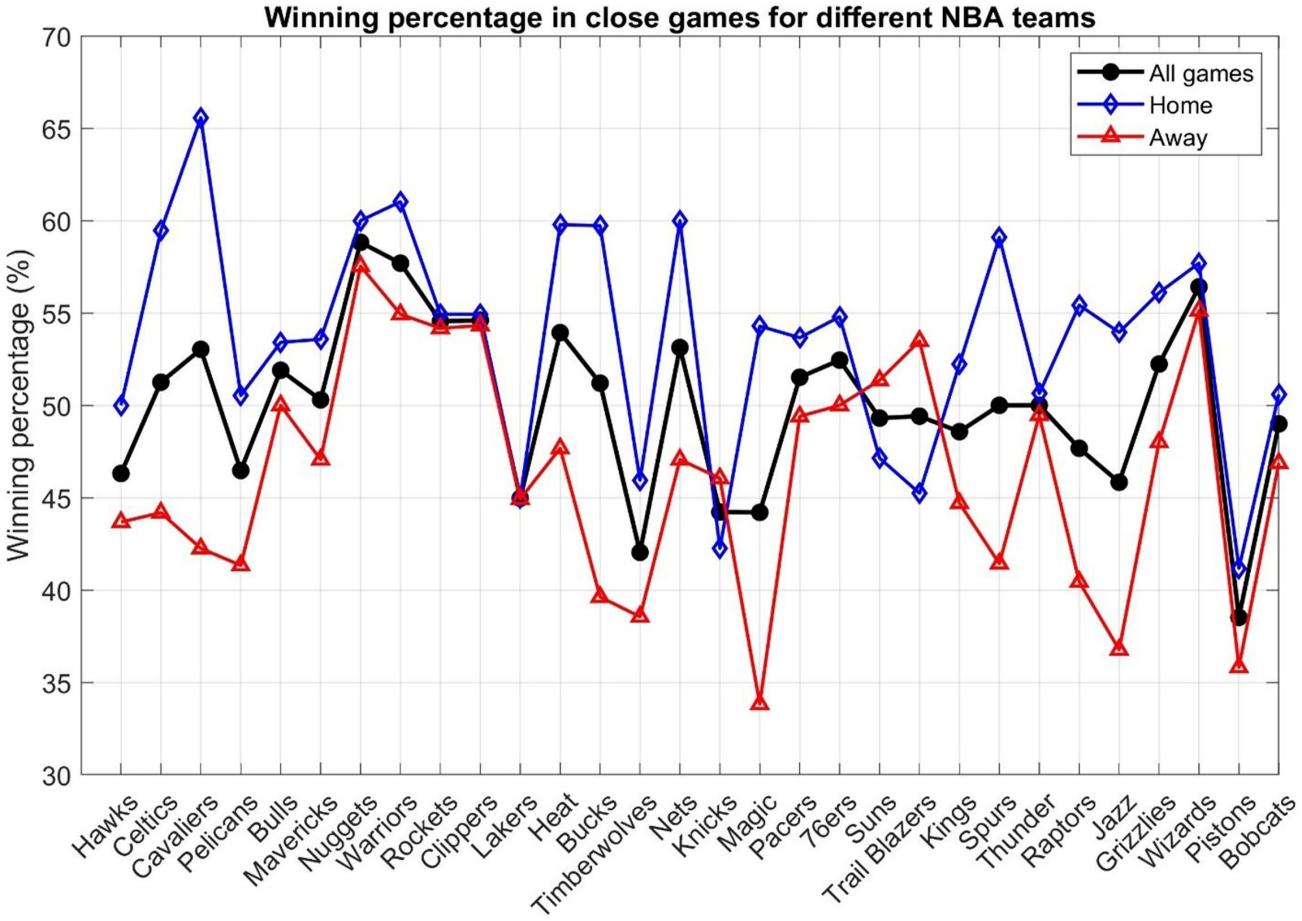
Winning percentage of all close games, as well as close games when played at home courts or on the road, for different NBA teams.

Figure 3 illustrates the winning percentages for all NBA teams in close games, both at home and away. A clear observation is that most teams exhibit a winning percentage ranging from 45 to 55% in these tightly contested matches. The Denver Nuggets lead the pack with a 58.8% win rate, followed by the Golden State Warriors at 57.7%, and the Washington Wizards at 56.4%. A noticeable disparity is evident in teams’ performances at home versus on the road. The majority boast a winning percentage north of 50% in their home games, underscoring the pivotal role the home court advantage plays in their success. Teams generally grapple with diminished winning percentages on the road. The 40–50% winning bracket is densely populated in this context, punctuated by notable exceptions like the Nuggets, Warriors, and Wizards, who transcend this bracket and boast over 55% winning percentages in close away games. The Orlando Magic, however, are anchored at the base with a win rate shy of 35%.

**Figure 4:**
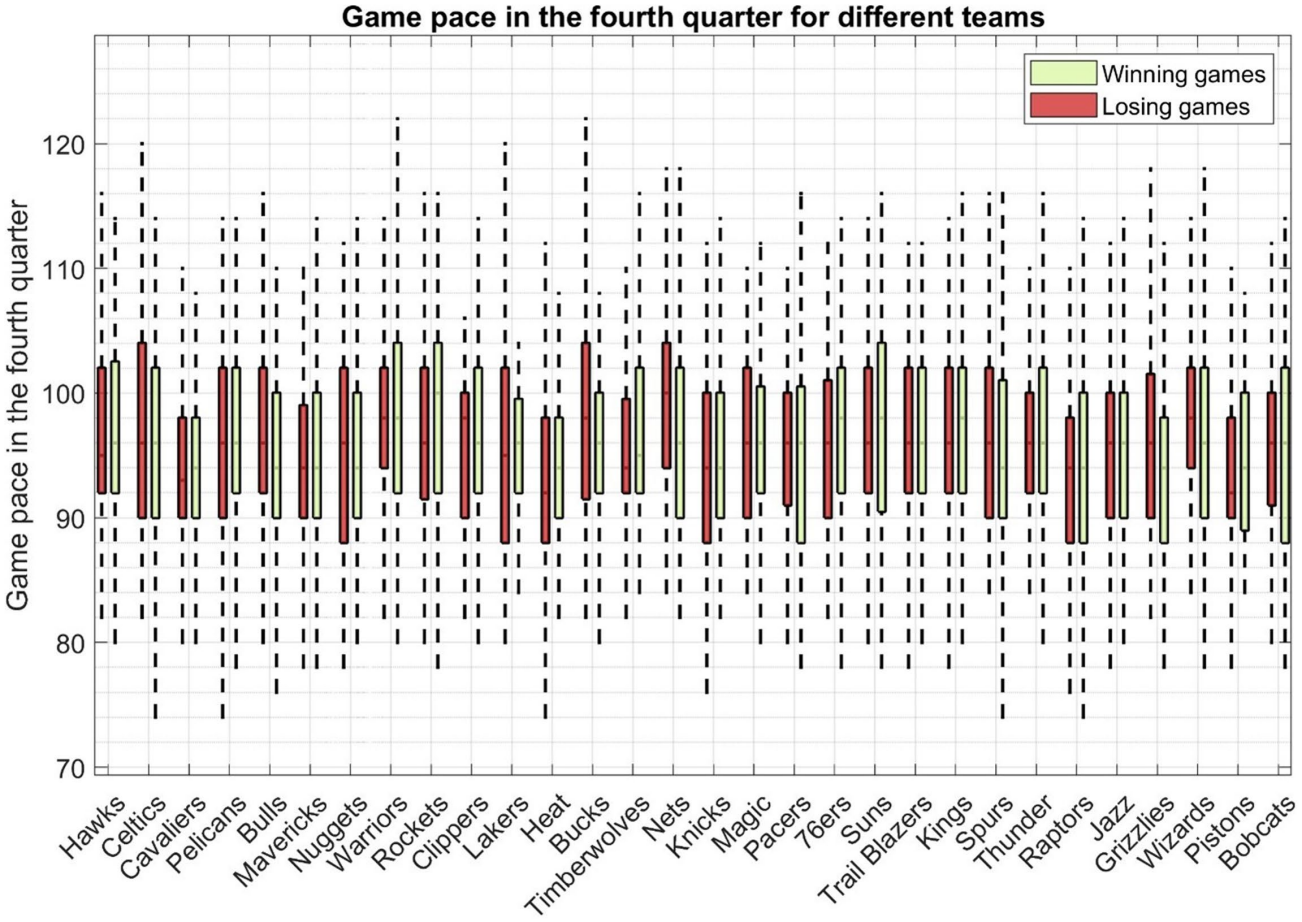
The pace of all the fourth quarters in closely contested basketball games across all teams. Each boxplot represents the pace calculated from both victorious and defeated matchups of a specific team.

Figure 4 illustrates the tempo of fourth-quarter play in closely contested basketball games across all teams. Each boxplot represents the pace, calculated from both victorious and defeated matchups of a specific team. The pace factor quantifies the number of possessions a team has during a basketball game. A couple of noteworthy observations can be made from this figure. Firstly, the median pace (50th percentile) for the boxplots consistently falls within the range of 90–100, demonstrating variations across different teams. In terms of losing games, the Nets exhibit the highest median pace, standing at 100, whereas both the Pistons and the Heat maintain a median pace of 92. Conversely, in winning games, the Rockets achieve the highest median pace, reaching 100, surpassing all other teams. Secondly, an intriguing insight emerges from the data: even for the same team, there exists a disparity between the pace of winning and losing games. For instance, consider the case of the team Houston Rockets, where the median pace for winning games stands at 100, in contrast to a median pace of 96 for losing games. This discrepancy suggests that a faster game pace may be advantageous for this team’s chances of victory. Conversely, this dynamic varies for some other teams. For instance, the Nets’ median pace for winning games is 96, while it rises to 100 in losing games. This contrast in game pace between winning and losing encounters reflects the distinct playing styles and individual player characteristics that shape a team’s performance.

**Figure 5:**
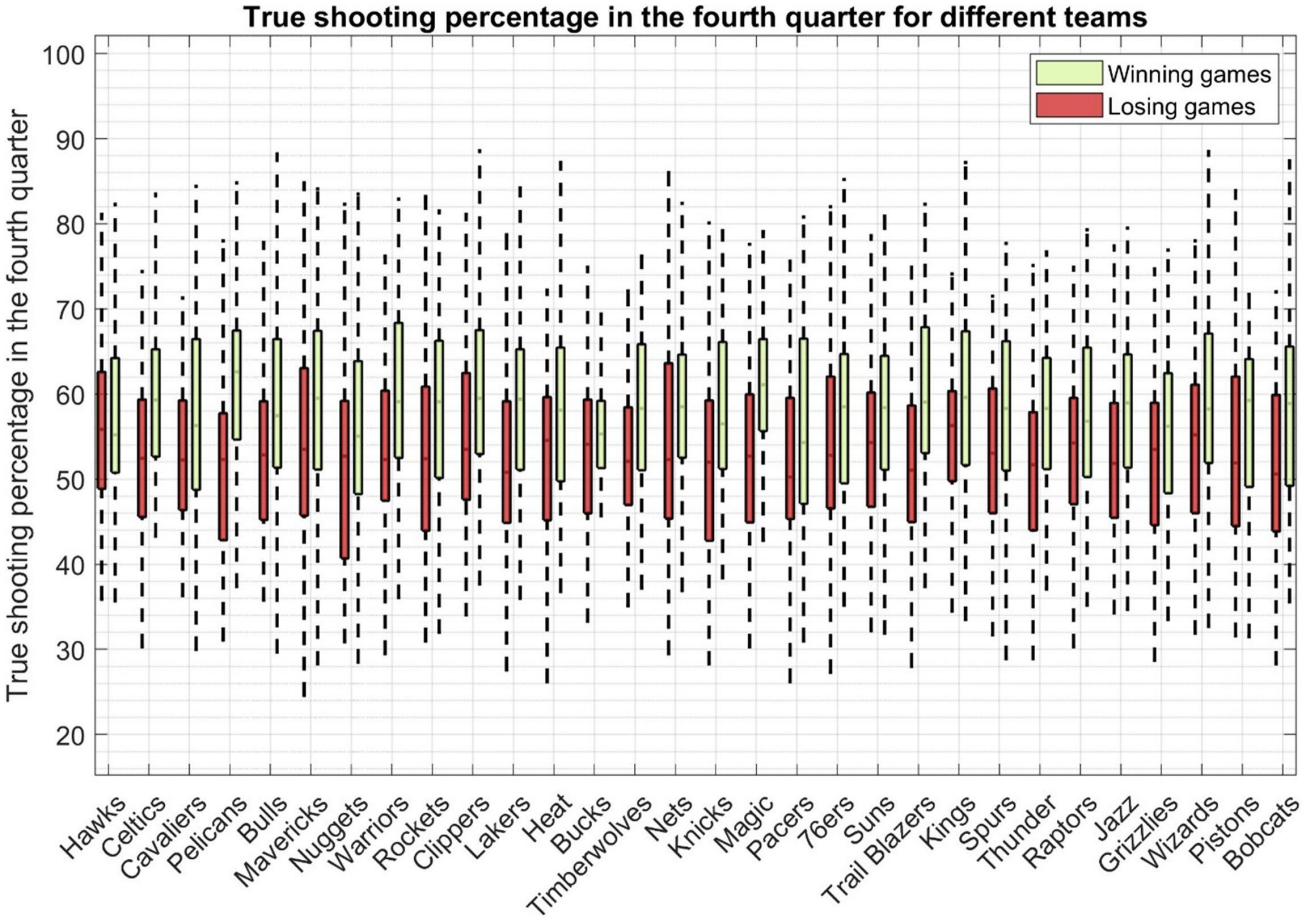
The true shooting percentage of all fourth quarters in closely contested basketball games across all teams. Each boxplot represents the true shooting percentage calculated from both victorious and defeated matchups of a specific team.

Figure 5 shows True Shooting Percentage (TS%) in the fourth-quarter play in closely contested basketball games across all teams. It is a valuable advanced statistic in basketball that takes into account not only field goals but also three-pointers and free throws. It’s an excellent indicator of a team’s scoring efficiency. A few observations can be drawn from this Figure. First of all, as expected, the true shooting percentage in winning games is higher than that of the losing games in close matchups except for one team, i.e., the Hawks. For the team Hawks, the statistic for its winning games is 55.20, while it is 55.8 for its losing games. This shows that the team is nearly consistent in close matchups, whether it ended up winning or losing the game. It also indicates that the team relies more on defense rather than offense in the fourth quarter in winning a close matchup. For the rest of the teams, true shooting percentage in winning games is higher than that in losing matchups. The difference could be up to as high as 10 percent for the team Pelicans. Similarly, for the team Bobcats, the median true shooting percentage for losing games is 50.6%, while it is 58.9% for winning games.

**Figure 6:**
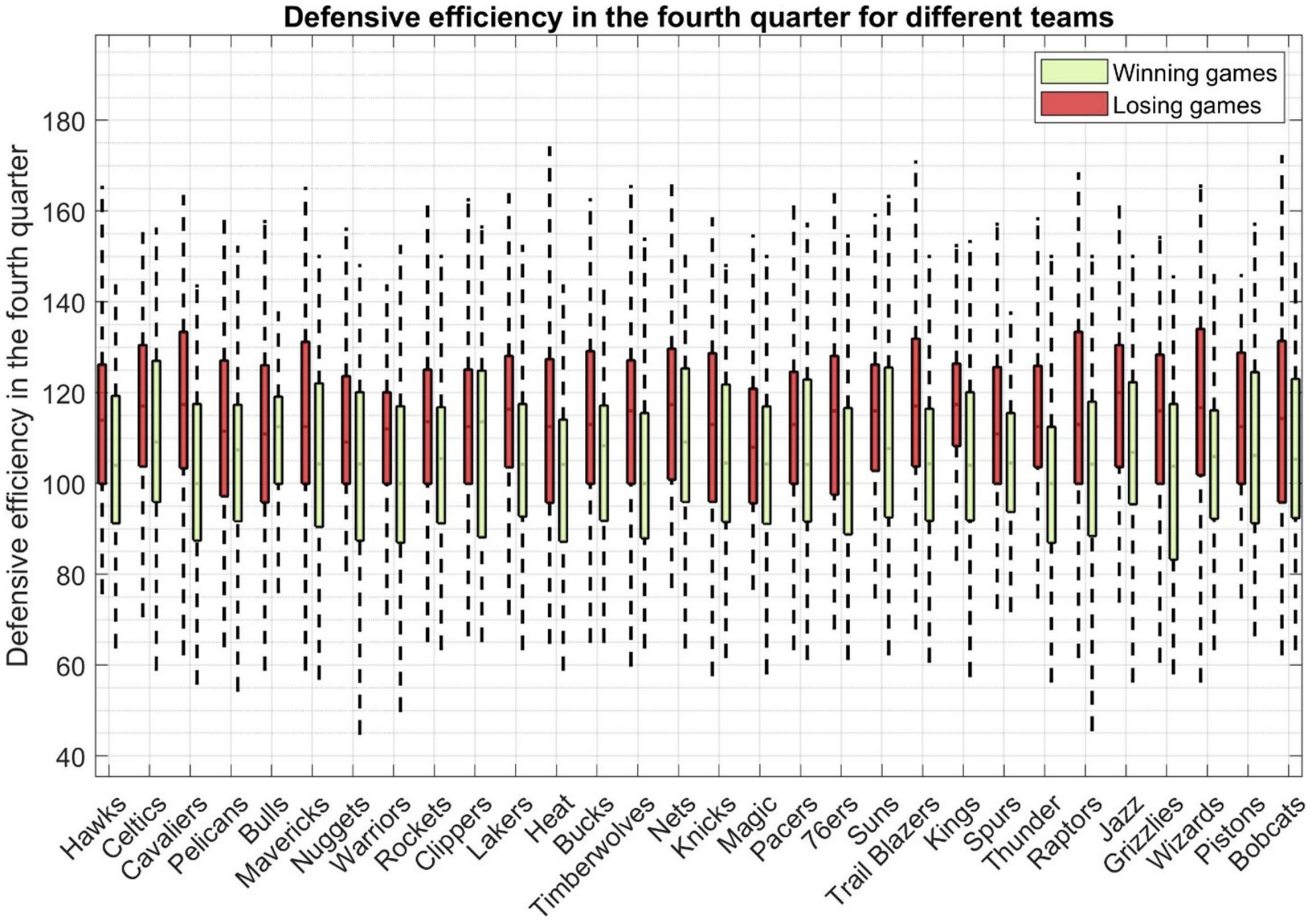
The defensive efficiency of all fourth quarters in closely contested basketball games across all teams. Each boxplot represents the defensive efficiency calculated from both victorious and defeated matchups of a specific team.

Defensive efficiency serves as a pivotal gauge of a team’s overall defensive performance. This metric, defined as the number of points allowed by a team’s opponent per 100 possessions, effectively neutralizes the impact of pace variations in the fourth quarter of closely contested games. As illustrated in Figure 6, we present the defensive efficiency data for the fourth quarter in close matchups across all teams. For the majority of teams, defensive efficiency tends to be lower in victorious games compared to their statistics in losing games.

Nonetheless, there are a few outliers to consider, most notably the Chicago Bulls and the Los Angeles Clippers. Interestingly, these two teams exhibit a minimal difference in defensive efficiency between their winning and losing games. Worth noting is that, based on fourth-quarter statistics, the Orlando Magic boasts the lowest defensive efficiency at 108 in its losing games.

### 3.2 Bayesian logistic modeling for the Houston Rockets

We employed data from the team Houston Rockets to assess the efficacy of the proposed method. This dataset encompasses 143 closely contested matchups, consisting of 65 losses and 78 wins. To establish the model’s parameters, 80% of these 143 games, totaling 115 matches, were utilized. The remaining 20% of the games, precisely 14 wins and 14 losses, were reserved for evaluating the model’s performance.

**Figure 7:**
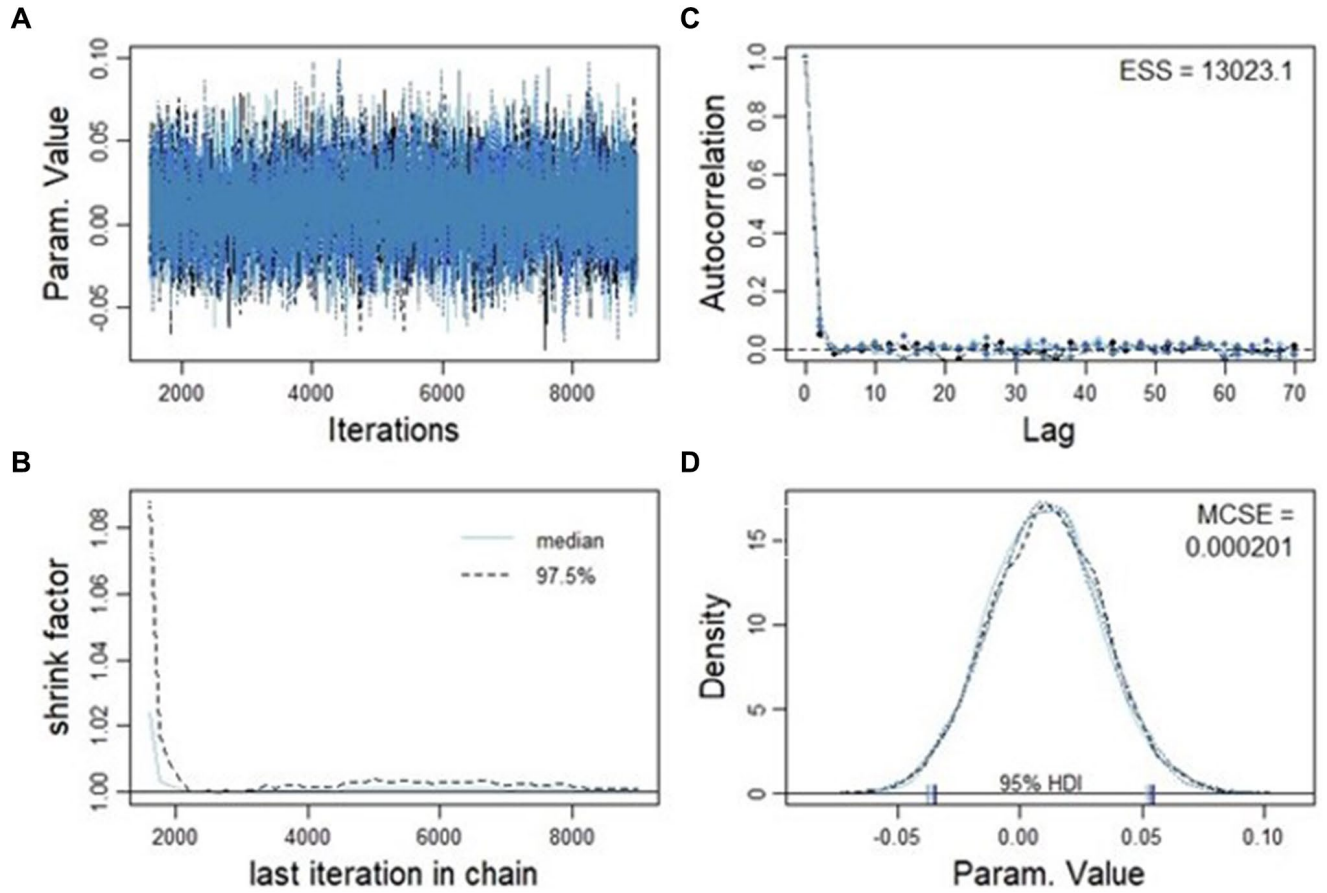
Convergence diagnostics for the MCMC chains. Panel **(A)** displays the trace plot of the four MCMC chains with no sign of orphaned chain; panel **(B)** shows the autocorrelations at different lags, which are nearly zero; panel **(C)** shows the Gelman-Rubin statistics that is close to 1.0, as an indication of chain convergence; panel **(D)** displays the comparison of estimated probability density curves from different chains. The minimal difference between the curves indicates that estimated probability density curves are consistent with each other.

Figure 7 presents various aspects of the analysis. Firstly, in Figure 7A, the trace plot of the four distinct Markov Chain Monte Carlo (MCMC) chains is displayed. These chains are overlaid to visually assess their convergence. The substantial overlap among the chains in Figure 7A provides a compelling indication that convergence has been achieved. Moving on to Figure 7B, attention is directed toward the Gelman-Rubin statistics, commonly referred to as the shrink factor. This metric assesses convergence by examining the ratio of between-chain variance to within-chain variance. As observed in Figure 7B, the shrink factor is in proximity to unity, a strong indicator that the MCMC chains have indeed converged satisfactorily. Figure 7C focuses on the effective sample size, accounting for autocorrelation within the MCMC chains. The small values of autocorrelation at various lags suggest that effective samples have been generated to construct the MCMC chains. Lastly, Figure 7D showcases smoothed density plots derived from distinct chains. Notably, these density plots exhibit minimal variation, as reflected in the Monte Carlo Standard Error (MCSE). This stability in density plots further bolsters confidence in the convergence and reliability of the analysis.

**Figure 8:**
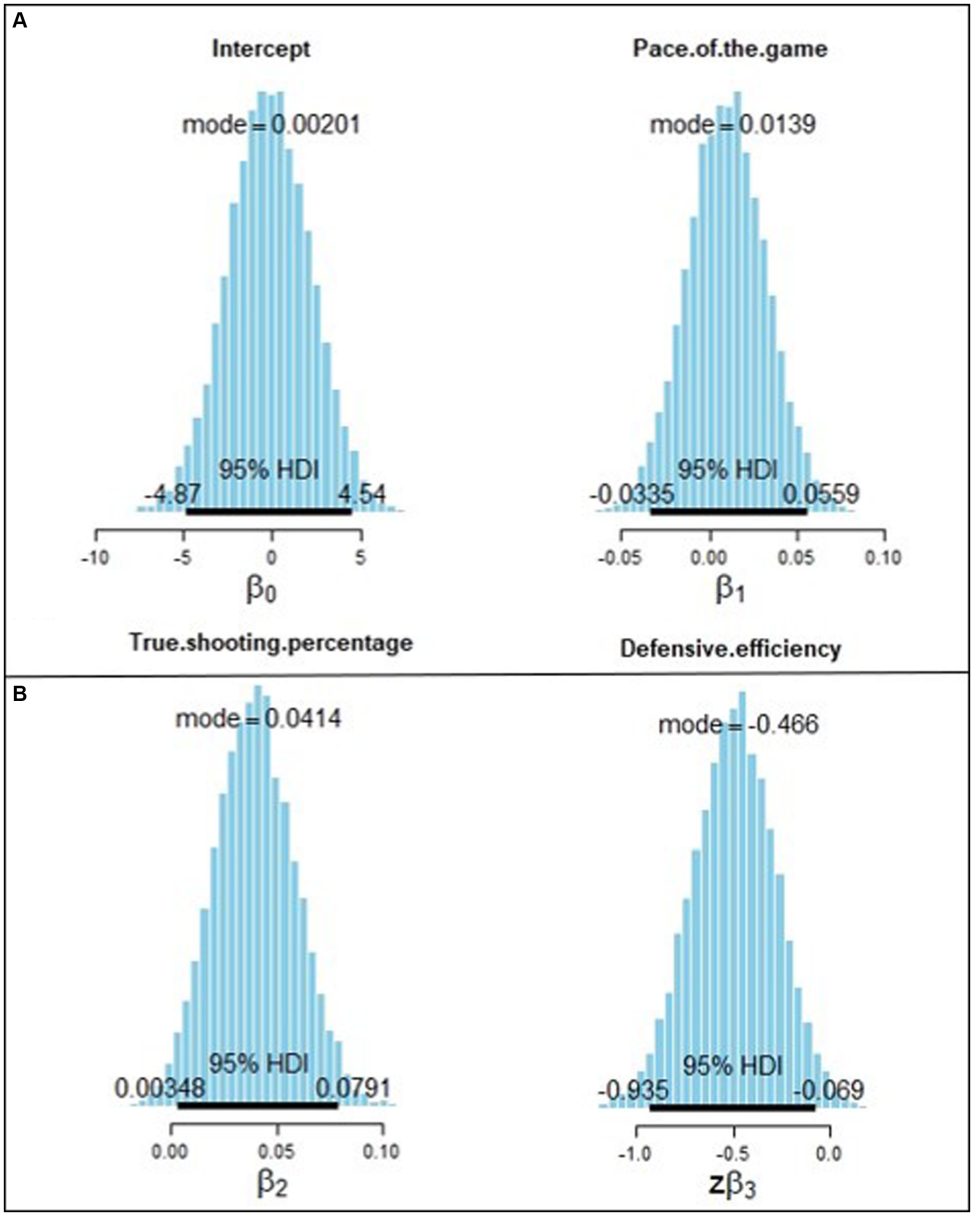
Posterior distribution of the four model parameters estimated from Markov Chain Monte Carolo (MCMC) chains. The model and the 95% of high-density interval (HDI) are shown for each parameter.

Figure 8 shows the estimated posterior distributions of model parameters, including the mode and the 95% high-density interval (HDI). All of the four parameters follow the normal distribution. The mode of the intercept parameter is 0.002; the 95% HDI ranges from −4.87 to 4.54. The mode of the parameter associated with true shooting percentage is −0.041; the 95% HDI ranges from 0.003 to 0.079. The magnitude of the model parameter associated with defensive efficiency is the largest, with a mode value of −0.466.

**Figure 9:**
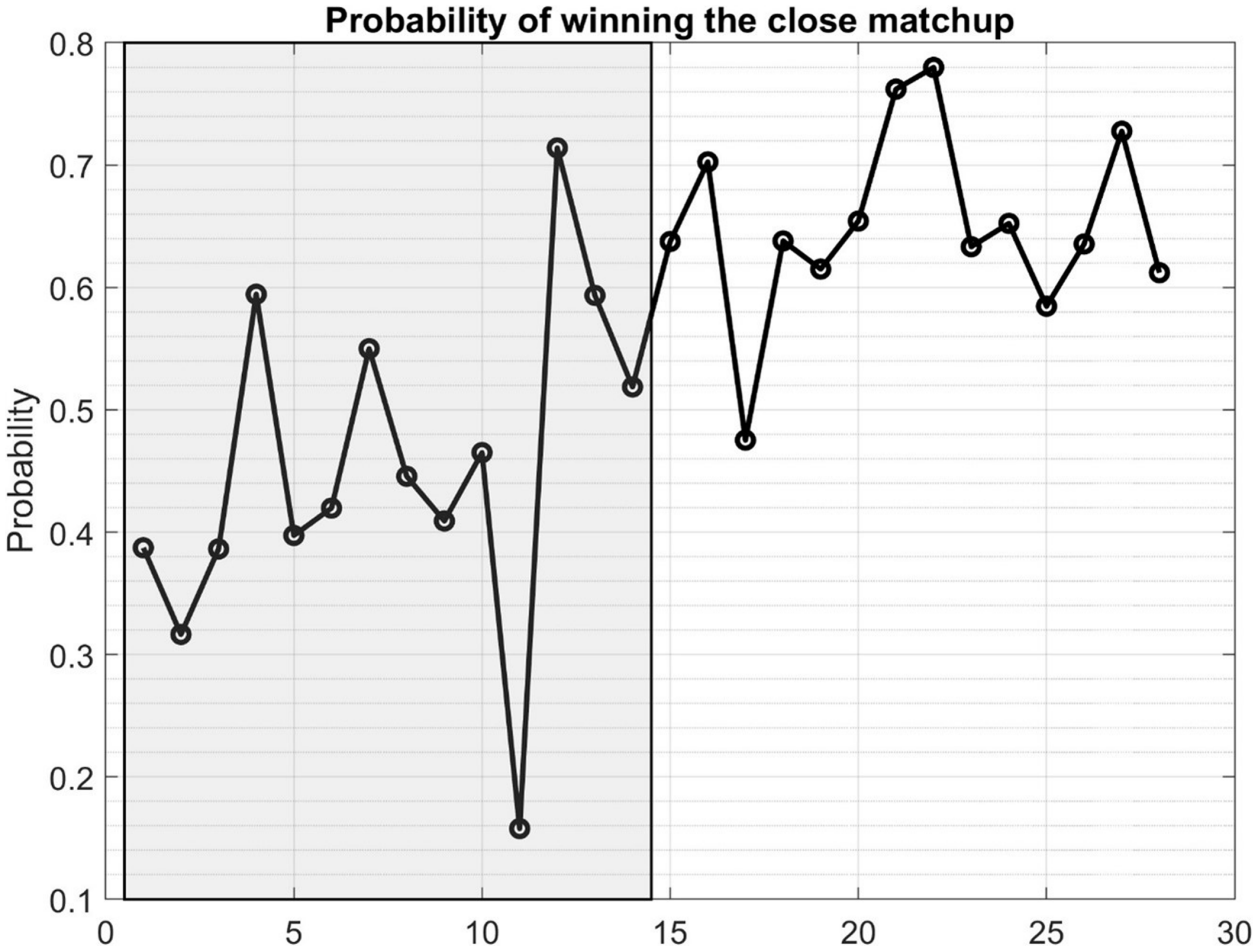
The mean probability of winning the games in the validation dataset for the team Houston Rockets, based on the Bayesian Logistic Model. The first 14 games with shade were losing matchups for the team and the remaining 14 games (non-shaded) were victorious games for the team.

Figure 9 illustrates the mean probability of winning across 28 validation games. Notably, the initial 14 games, marked within the shaded region, represent losses for the Houston Rockets, while the subsequent games correspond to victories. This visual depiction underscores an overall trend where the estimated winning probability tends to be lower for the losing games. If a threshold value of 0.6 is selected, among the 14 losing matchups, the estimated winning probability falls below the identified threshold in 13 instances. For only 1 out of the 14 losing matchups, the model suggested winning chance is greater than 0.6. In sharp contrast, among the 14 winning games, the estimated winning probability exceeds 0.6 in 13 out of the 14 games. True Positive Rate of this model is hence 0.93 (13 out of 14 winning games) and False Positive Rate is 0.07 (1 out of 14 losing games).

In comparison with the regression model, the team’s winning probability (0.545) in historical years is used as the baseline model. Brier score of the fitted logistic regression model is 0.18. while the Brier score of the baseline model is 0.25, which is higher than the proposed method. This indicate the proposed method can better predict game outcomes than the baseline model. This is also reflected in the Brier Skill score, the value of which is 0.28.

**Figure 10:**
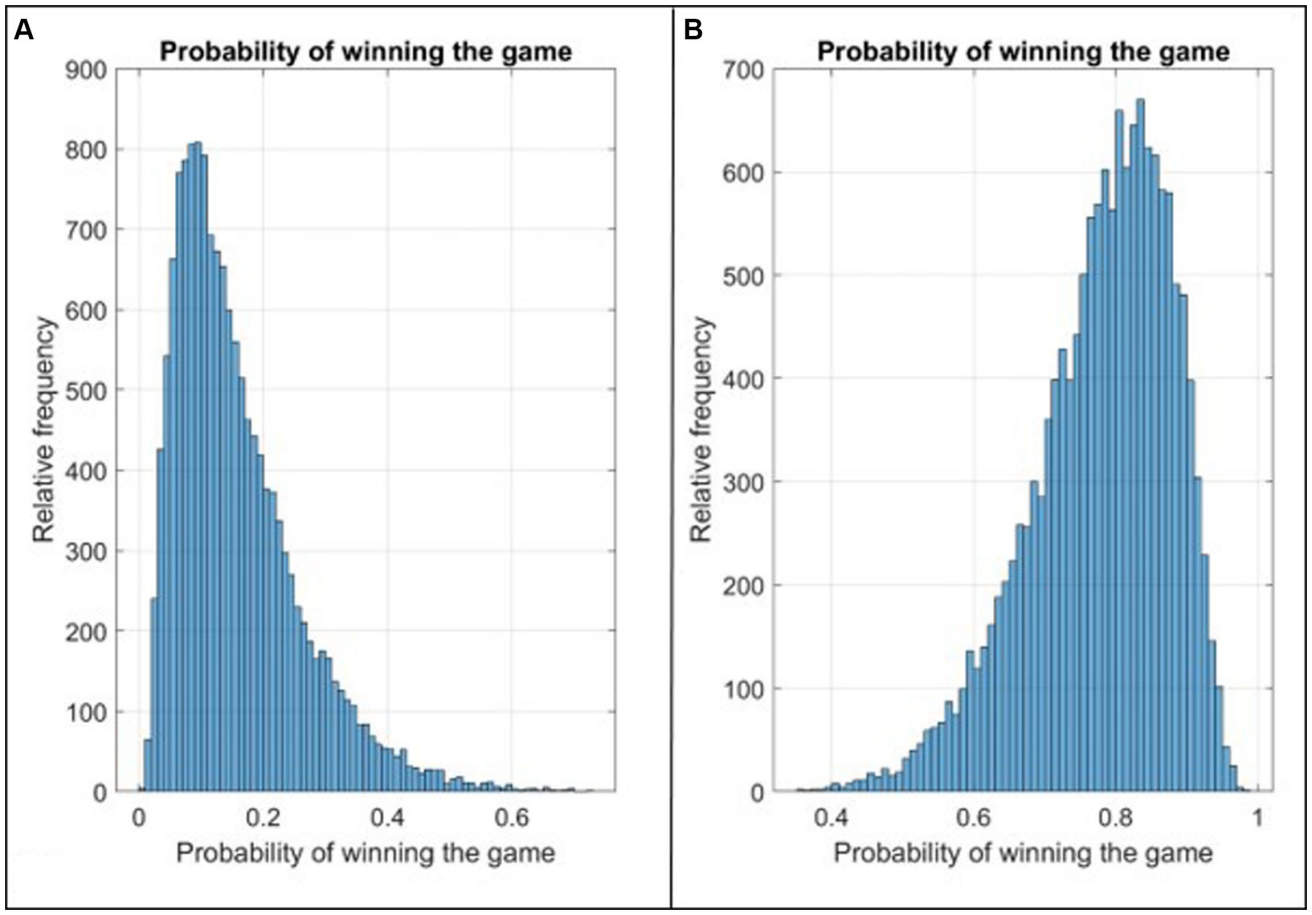
**(A)** The relative frequency of winning probability calculated from the Logistic Regression Model for the defeated game with the lowest probability in Figure 9; **(B)** The relative frequency of winning probability calculated from the Logistic Regression Model for the victorious game with the highest probability in Figure 9.

Figure 10 presents two instructive illustrations, each corresponding to one of the victorious or defeated games depicted in Figure 9. In the case of the game ending in defeat, the fourth-quarter pace was measured at 92, with a true shooting percentage of 33.9%, and a defensive efficiency score of 160.9. Figure 10A displays a histogram illustrating the estimated probabilities of winning the game, derived from the fitted logistic regression model, with mean and median values of 0.13 and 0.16, respectively.

Contrastingly, for the victorious game featuring the highest probability of winning among those in Figure 9, the respective values of its three predictive variables - fourth-quarter pace, true shooting percentage, and defensive efficiency – stood at 94, 81.5%, and 108.7. Figure 10B showcases a histogram representing the probability estimates obtained from the fitted logistic regression model for this winning game. Here, the median and mean values of the winning probabilities are 0.78 and 0.79, respectively.

## 4 Discussion

Understanding key performance indicators (KPI) in determining the outcome of close basketball games is of paramount importance for teams, players, coaches, and analysts alike. These KPI serve as the compass guiding decision-making, strategic adjustments, and player development in high-pressure moments. By identifying and prioritizing critical aspects of the game, such as shot selection, defensive strategies, and clock management, teams can make informed decisions on the court. The fourth quarter or even the last 5 min of the games requires players to perform under pressure (Wallace et al., 2013; Solomonov et al., 2015; Cabarkapa et al., 2022). Results in this study confirms that the shooting percentage is a KPI, playing an important role in determining the outcome of the games. Çene (2018) found that the quality of shots are more important than the quantity of shorts in close basketball games, by examining the basketball matches in the 2016–2017 Euroleague season. Gomez et al. (2016) analyzed 48 mean’s NBA close games during the 2013–14 regular season and found that starting quarter score, ball possession success, and defense are important variables in the dynamics during the 4th game quarter of NBA close game. The authors, however, did not use the game score at the beginning of the 4th quarter to identify the close games.

For coaches and team strategists, the model offers insights, illuminating potential tactical pivots that could tilt the game in their favor. A meticulous analysis of the ongoing game pace can inform adjustments to either accelerate offensive onslaughts or slow down the game to disrupt the opponents’ rhythm. The true shooting percentage provides a granular view of players’ performance under pressure, guiding decisions on who should take the crucial shots. Defensive efficiency stats, meanwhile, become pivotal in orchestrating defensive formations and substitutions, zeroing in on optimizing the team’s resilience against the opponent’s offensive forays.

This study is not without limitations, and it is essential to acknowledge that the scope of KPI in high-pressure moments in the game transcends tactical parameters and deeply intertwines with the psychological preparedness of players and teams. In moments of heightened tension, such as buzzer-beaters or pivotal free throws, the mental facets of the game ascend in significance. A player’s ability to manage anxiety, foster self-assurance, and uphold composure under pressure is crucial, marking a dimension that extends beyond mere statistics. Schweickle et al. (2023) underscore the importance of athletes’ subjective experiences and perceptions of their performance in high-pressure scenarios. These subjective aspects often escape the quantitative grasp of traditional performance indicators and statistics, highlighting a gap that warrants attention. The inner psychological battleground where confidence and anxiety clash, especially during critical moments, is pivotal in shaping outcomes but remains elusive in the present study due to data constraints. Another limitation of this study is that the model does not explicitly account for personnel changes, including players and coaches and game locations. Although the playing style can reflect in the pace of the game, consideration of external variables such as coaching style, game location, and traveling and back-to-back games (Charest et al., 2021; Esteves et al., 2021) may further enhance the model.

Therefore, while this study offers valuable insights, further research can be done and there is a recognized need for comprehensive research that melds objective performance metrics with the often intangible yet impactful psychological and atmospheric elements of the game. Expanding the analytical lens to encapsulate these dimensions promises a more holistic understanding of performance in high pressure situations, offering a richer tapestry of insights that span the statistical, psychological, and experiential facets of basketball.

## 5 Conclusion

In this study, relevant game statistics from 2,295 close games in the past decade, ranging from 2013–14 season to 2022–23 season, were obtained. A closely contested game is characterized by a margin of fewer than 10 points as the fourth quarter begins, and the ultimate victor emerges with a lead of less than 5 points by the end of the fourth quarter. To facilitate data retrieving, the NBA application programming interface (API) was used to download data.

Most teams exhibit a winning percentage ranging from 45 to 55% in these tightly contested matches. A noticeable disparity is evident in teams’ performances at home versus on the road. The majority boast a winning percentage north of 50% in their home games, underscoring the pivotal role the home court advantage plays in their success. Favorable game pace varies among the teams. This contrast in game pace between winning and losing encounters reflects the distinct playing styles and individual player characteristics that shape a team’s performance. True Shooting Percentage (TS%) in the fourth-quarter play in closely contested basketball games is an important variable, as an excellent indicator of a team’s scoring efficiency. As expected, the true shooting percentage in winning games is higher than that of the losing games in close matchups. Defensive efficiency serves as a pivotal gauge of a team’s overall defensive performance, exerting a decisive influence on the outcome of closely contested matchups. For the majority of teams, defensive efficiency tends to be lower in victorious games compared to their statistics in losing games, allowing lower points from the opponent team.

A robust Bayesian logistic regression model that incorporates team performance statistics during the 4th quarter was proposed in this study. The binary outcome of the close game is modeled as a Bernoulli distribution. The posterior distribution of model parameters was derived by employing the Markov Chain Monte Carlo (MCMC) technique, utilizing the Gibbs sampling algorithm. The model was tested using the data from the team Houston Rockets and relevant performance metrics were used to evaluate its performance. True Positive Rate and False Positive Rate is 0.93 and 0.07, respectively. The practical implications are also discussed. Most importantly, for coaches and team strategists, the model offers real-time insights, illuminating potential tactical pivots that could tilt the game in their favor.

## Data availability statement

The original contributions presented in the study are included in the article/supplementary material, further inquiries can be directed to the corresponding author.

## Author contributions

FW: Conceptualization, Data curation, Formal analysis, Investigation, Methodology, Validation, Visualization, Writing – original draft, Writing – review & editing. GZ: Conceptualization, Supervision, Writing – review & editing. LH: Writing – review & editing, Data curation, Visualization.

## Footnotes

^1^ https://www.nba.com/stats/

^2^ https://github.com/swar/nba_api
